# COVID-19 Vaccination in Cancer Patients Older Than 70 Years Undergoing Active Treatment. Seroconversion Rate and Safety

**DOI:** 10.3390/vaccines10020164

**Published:** 2022-01-21

**Authors:** Luigi Cavanna, Manuela Proietto, Chiara Citterio, Elisa Anselmi, Elena Zaffignani, Elisa Maria Stroppa, Maria Teresa Borsotti, Andrea Contini, Gabriella Di Girolamo, Vincenzo Matteo Quitadamo, Nicoletta Bacchetta, Monica Muroni, Maria Grazia Brescia, Marco Delledonne

**Affiliations:** 1 Oncology and Hematology Department, Piacenza General Hospital, Via Taverna 49, 29121 Piacenza, Italy; m.proietto@ausl.pc.it (M.P.); c.citterio@ausl.pc.it (C.C.); e.anselmi@ausl.pc.it (E.A.); e.zaffignani@ausl.pc.it (E.Z.); e.stroppa@ausl.pc.it (E.M.S.); m.borsotti@ausl.pc.it (M.T.B.); v.quitadamo@ausl.pc.it (V.M.Q.); n.bacchetta@ausl.pc.it (N.B.); m.muroni@ausl.pc.it (M.M.); 2 Nurse Directory Unit, Piacenza General Hospital, Via Taverna 49, 29121 Piacenza, Italy; a.contini@ausl.pc.it (A.C.); g.digirolamo@ausl.pc.it (G.D.G.); 3 Public Health Unit, Piacenza General Hospital, Via Taverna 49, 29121 Piacenza, Italy; m.brescia@ausl.pc.it (M.G.B.); M.DELLEDONNE@ausl.pc.it (M.D.)

**Keywords:** COVID-19, SARS-CoV-2, vaccine, cancer patients, old patients

## Abstract

Patients with cancer have a high risk of intubation, intensive care unit admission, or death from the coronavirus disease (COVID-19); age and comorbidities are additional risk factors. Vaccination is effective against COVID-19; however, patients with cancer have been excluded from pivotal clinical trials for COVID-19 vaccines. Data on COVID-19 vaccination in cancer patients who are older are lacking. This observational study was conducted to evaluate the seropositivity rate and safety of a two-dose regimen of the BNT162b2 or mRNA1273 vaccine in older patients (age ≥ 70 years) with solid tumors or with hematological malignances who are undergoing active anticancer treatment or whose treatment has been terminated within 6 months of vaccination. The control group was composed of healthy volunteers that were age-matched with the patient group. The primary endpoint was the seropositivity rate, and the secondary endpoints were safety, the factors influencing seroconversion, the IgG titers of patients versus healthy volunteers, and post-vaccine COVID-19 infection between 20 March 2021 and 14 July 2021. At our Institution (Oncology and Hematology Department, Hospital of Piacenza, North Italy), 443 patients with cancer underwent a program for COVID-19 vaccination; 115 (25.95%) were older than 70 (range 71–86 years) and form the basis of this study. All 115 patients accepted the vaccination. There were 64 female patients (55.65%), 94 patients (81.74%) with solid tumors, and 21 patients (18.26%) with hematological malignances. The primary endpoint of seropositivity was observed in 75 patients (65.22%)—70.21% in patients with solid tumors and 42.86% in patients with hematological malignances—versus in 100% of patients in the control group. Of the secondary endpoints, no grade 3–4 side effects and no COVID-19 infections were reported. The factor influencing seroconversion was the type of cancer. The patients’ median IgG titers were significantly lower than in the control groups. The COVID-19 vaccines BNT162b2 and mRNA1273 were effective and safe among older patients with cancer when administered in real-world conditions.

## 1. Introduction

A novel coronavirus named severe acute respiratory syndrome coronavirus 2 (SARS-CoV-2) emerged in Wuhan, China, in December 2019 and has quickly spread globally [1,2,3]. SARS-CoV-2 is a highly contagious respiratory pathogen that causes a disease that has been termed the 2019 coronavirus disease (COVID-19). Clinical experience indicates that COVID-19 is highly heterogeneous, ranging from being asymptomatic and mild to severe and deadly [4,5]. Cancer patients are at a high risk of acquiring this virus because of poor general conditions, a systemic immunosuppressive state caused by cancer itself, and/or anticancer treatment [6,7,8].

Early data from China demonstrate that the case fatality ratio (CFR) of COVID-19 increases with age, ranging from 0.4% or lower in patients aged <50 years, 1.3% among those aged between 50 and 60 years, 3.6% among those aged between 60 and 70 years, 8% among those aged between 70 and 80 years, to 14.8% among those older than 80 years [9]. A more profound effect of aging is shown by COVID-19 CFR data from Italy, the first country affected by the pandemic after China. Again, CFRs range from less than 0.4% in patients aged <50 years, 1% among those aged between 50 and 60 years, 3.5% among those aged between 60 and 70 years, 12.8% among those aged between 70 and 80 years, to 20.2% among those older than 80 years [10]. Aging is an important risk factor for severe COVID-19 and its adverse health outcomes, including hospitalization, intensive care unit (ICU) admission, and death [11]. In addition, patients with cancer and COVID-19 have a marked elevated risk of intubation and death, whether these patients are receiving active anticancer treatment or are cancer survivors [12].

We previously reported cases involving the first 25 cancer patients with COVID-19 pneumonia in the Western world, and we found a mortality rate of 36.00% [3]. Additionally, cases involving 51 cancer patients with COVID-19 were reported by our group, and we found a COVID-19-related mortality rate of 23.53% [6]. In addition, advanced age was one of major risk factor for mortality. Vaccines (BNT162b2 and mRNA-1273) were approved and recommended by the United States Food and Drug Administration (FDA) and the European Medicines Agency to prevent COVID-19 [13]. BNT162b2 and mRNA-1273 are lipid nanoparticle-encapsulated messenger RNA (mRNA)-based vaccines [13,14]. However, patients with cancer have been excluded from pivotal clinical trials for COVID-19 vaccines, and there is a paucity of data on the efficacy and safety of mRNA COVID-19 vaccines in cancer patients [15,16,17,18,19].

Considering that cancer patients who are older are a very frail group at a high risk of contracting COVID-19, vaccination represents a cornerstone in preventing a catastrophic evolution of the disease in this subset of patients, and it is paramount in clinical practice to establish the efficacy and safety of vaccinations against COVID-19 in cancer patients older than 70 years. In the present report, we analyze in a prospective observational study the efficacy of COVID-19 vaccines, evaluating the antibody response and safety in cancer patients older than 70 years who are attending the oncology/hematology department at the Hospital of Piacenza.

## 2. Materials and Methods

This observational study was conducted at the Oncology-Hematology Department of Piacenza General Hospital (North Italy) to investigate the immunogenicity of COVID-19 vaccines in a prospective study that was approved by the local Ethics Committee (Institutional Review Board approval number 317/2021/OSS/ASLPC). COVID-19 vaccination was proposed by oncologists and trained nurses to all cancer patients attending the inpatient and outpatient clinics of the oncology-hematology department at the Hospital of Piacenza. All patients gave signed, informed consent. Patients were vaccinated at the Oncology-Hematology Department of Piacenza General Hospital through national and regional Italian programs. All participants were given the two-dose regimen of the BNT162b2 mRNA vaccine (Pfizer–BioNTech) or the mRNA-1273 vaccine (Moderna) via intramuscular injection in the deltoid muscle, according to the manufacturer’s technical instructions. Current treatment groups consisted of chemotherapy, hormone therapy, biological therapy, and immunotherapy, alone or in different combinations. We pooled treatments into five groups to facilitate comparisons: chemotherapy, biological therapy, immunotherapy, hormone therapy, and no treatment. This study also included a control group of healthy volunteers who were age-matched with the patient group undergoing COVID-19 vaccination during the same period.

### 2.1. Times of Vaccination

The vaccine was administered to patients undergoing cytotoxic chemotherapy 1–2 weeks before or 1–2 weeks after their drug dose. The vaccine was administered to patients treated with biological therapy (such as monoclonal antibodies and tyrosine kinase inhibitors), hormone therapy, or immune checkpoint inhibitors when available, as recommended [20]. For all cancer patients undergoing anticancer treatment, a complete blood cell count was conducted before vaccination in this observational study, and vaccination was delayed until absolute neutrophil count recovery. Eligibility criteria for this study were as follows: cancer patients with a solid tumor or hematological malignancy, aged ≥70 years, on active anticancer treatment or whose treatment had been terminated up to 6 months before the study period, and with no known history of SARS-CoV-2 infection or who were at least 3 months after testing positive for COVID-19. The blood serum of cancer patients was tested to evaluate serum IgG antibody levels against SARS-CoV-2 up to two days before vaccination (T0) and 2–6 weeks after the administration of the second vaccine dose. Patients with an active COVID-19 infection, a baseline IgG value of ≥15 BAU/mL, or with ≤70 years were vaccinated but were excluded from this study.

### 2.2. Endpoints

The primary endpoint was the proportion of patients who acquired anti-SARS-CoV-2 antibodies after two doses of vaccination. The secondary endpoints were as follows:

- Safety;
- Factors influencing vaccine seropositivity;
- Diagnosis of symptomatic or asymptomatic post-vaccine COVID-19 infection;
- Median IgG titer of patients versus healthy volunteers.

All vaccinated patients were followed and/or treated for their oncologic disease, and a COVID-19 swab test was performed in our department, as previously reported [21,22], for each patient attending the Oncology Clinic. This was repeated in asymptomatic patients every month.

### 2.3. Safety of the Vaccine

All patients were instructed to call specialized nurses from the Oncology Department to report any adverse events related to the vaccination, and they completed a questionnaire to record side effects exhibited between 1, 2, and 4 weeks after the first and the second vaccination doses.

### 2.4. Serological Assessment

Serum samples were analyzed and evaluated for SARS-CoV-2 antibodies with LIAISON SARS-CoV-2 S1-S2 IgG [23], performed according to the manufacturer’s technical instructions, which uses an automated platform, LIAISON XL, for the detection of IgG against subunits S1 and S2 of the SARS-CoV-2 spike protein. The results were calculated by referring to a calibration curve and expressed in binding antibody units (AU)/mL. A value ≥15 AU/mL was considered positive, according to the manufacturer’s instructions.

### 2.5. Statistical Analysis

Patients were registered with a unique recognition code in a Microsoft Excel file (Microsoft Office version 2010). Quantitative variables were described by their median and their interquartile range (IQR), and qualitative variables were described by their absolute and relative frequencies. Comparisons of covariates were conducted using Pearson’s X^2^ test or Fisher’s exact test for categorical variables, and the nonparametric Mann–Whitney U test for continuous variables. Univariable and multivariable analyses were performed using logistic regression to examine the association of each predictor variable with the response status of the patient. For each risk factor, the odds ratio (OR) with associated confidence intervals has been presented. All analyses were performed using STATA16 statistical software with two-sided significance tests and a 5% significance level.

## 3. Results

Overall, 115 oncology patients aged ≥70 years attending the Oncology/Hematology Department of Piacenza General Hospital between 20 March 2021 and 14 July 2021 were included in the study. The median age was 73 (IQR, 72−76) years, 55.65% of participants were female, and the median body mass index (BMI) was 24.08 kg/m2 (Table 1). Fifty-eight healthy volunteers constituted the control group, the median age was 71 (IQR, 70−74) years, and 60.34% were female.

Most patients (65.22%) were metastatic and with solid tumors (81.74%); the most common solid tumor was gastrointestinal (32.98%). Overall, 100 patients (86.95%) were on active anticancer treatment, and the most common anticancer treatment was chemotherapy (57.39%). A total of 64.35% of patients had one or more comorbidities; the most common were hypertension (39.13%) and cardiovascular disease (24.35%). A total of 33.04% had other comorbidities (benign prostatic hyperplasia, respiratory disease, osteoporosis, etc.). The baseline laboratory parameters, evaluated within one week of the first vaccine dose, included neutrophil count (×103/μL) (median 3.15 (IQR 2.4–4.65)) and lymphocyte count (×103/μL) (1.45 (IQR 0.9–1.89)).

After the second dose, 75 patients (65.22%) had an IgG value of ≥15 AU/mL and 40 patients (34.78%) did not show a serologic response; seropositivity in the control group was 100%. The median IgG value at T1 was significantly higher in the seroconverted group (189 (IQR: 60–280) AU/mL vs. 3.8 (IQR: 3.80–5.55) AU/mL, *p*-value < 0.01) (Table 1). The lowest seroconversion rate was observed in patients with hematological malignances (42.86% seroconverted vs. 70.21% of patients with solid tumor, *p*-value 0.02). No significant differences in seroconversion were seen when comparing patients on active cancer therapy with patients who were not (64.00% versus 73.33%) or when comparing different treatments (chemotherapy 63.64%, immunotherapy 52.94%, biologic therapy 76.92%, hormone therapy 75.00%, and no treatment 73.33%); however, significantly lower rates of seropositivity were seen in patients treated with anti-CD-20 (Rituximab) (only 1 patient (14.29%) seroconverted, *p*-value 0.01). There was no significant association in multivariable analyses between seroconversion rate and age, BMI, solid tumor location, cancer stage, treatment, line of treatment, comorbidities, or laboratory parameters (data not shown).

**Figure 1:**
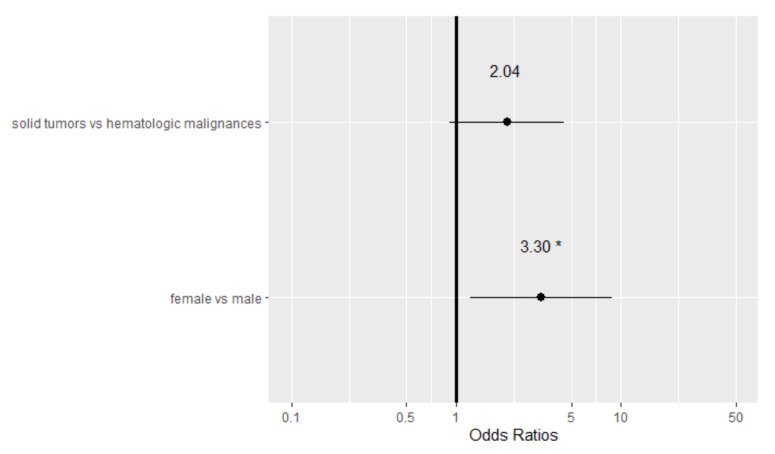
A forest plot of the odds ratios (OR) for seroconversion following vaccination from a multivariable logistic mode (* indicate *p*-value < 0.05).

**Figure 2:**
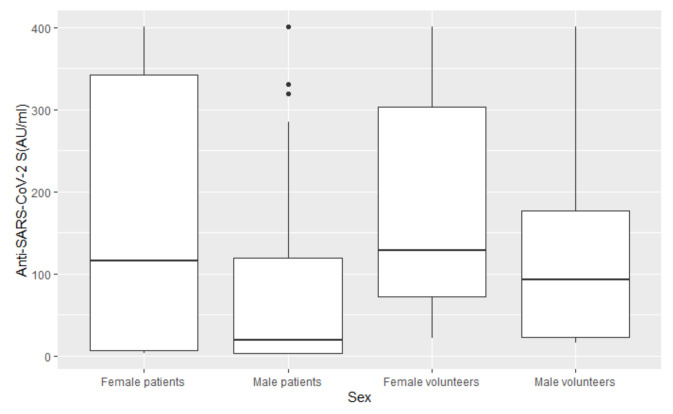
The IgG (AU/mL) values by sex of patients and healthy volunteers. (Anti-SARS-CoV-2 S: Anti-severe acute respiratory syndrome-associated coronavirus2 spike protein).

**Figure 3:**
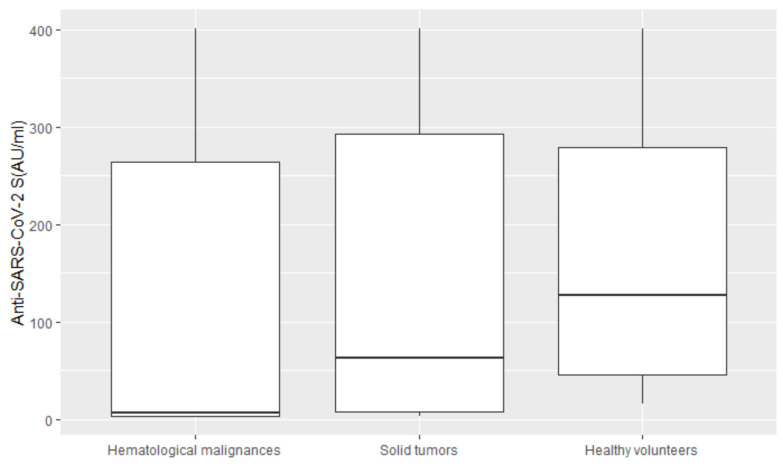
The IgG (AU/mL) values of patients with hematological malignances, patients with solid tumors, and healthy volunteers. (Anti-SARS-CoV-2 S: Anti-severe acute respiratory syndrome-associated coronavirus2 spike protein).

In a multivariable analysis (Figure 1), the only variable significantly associated with seroconversion was the type of cancer. We observed that the relationship between seroconversion and type of cancer remained significant after accounting for the effect of sex. Sex was marginally statistically significant in the univariable analysis (*p*-value 0.10) but evaluated as clinically relevant from data in the literature. The results from the multivariable logistic regression showed that patients with solid tumors have a higher probability of seroconversion after vaccination (OR 3.30 with a 95% confidence interval (CI) of 1.23–8.87, *p*-value 0.02) when compared to patients with hematological malignances. The IgG values (AU/mL) by sex of patients and healthy volunteers are reported in Figure 2, and those by cancer type are reported in Figure 3.

## 4. Discussion

COVID-19 is a heterogeneous infectious disease, and it is known that major risk factors for severe COVID-19 include age, male sex, obesity, smoking, and chronic comorbid conditions such as diabetes, cancer, and hypertension [3,6,9,11]. It must be emphasized that age is the most significant risk factor for severe COVID-19 and its adverse health outcomes, and it is known that older adults experience greater negative outcomes related to the COVID-19 pandemic when compared to younger patients [9,11]. These negative outcomes are amplified in older patients with COVID-19 and cancer [3,6,24,25], and it is well known that cancer is a major cause of mortality for older individuals [26]. In addition, the number of older adults with cancer is increasing rapidly. Older cancer patients have been underrepresented in cancer clinical trials, and cancer patients were excluded from the clinical trials of COVID-19 vaccines [27]. Therefore, data on the efficacy (evaluated by serologic response) and safety of COVID-19 vaccines in older patients with cancer who are undergoing anticancer treatment or whose treatment terminated within 6 months of vaccination are limited [15,16,17,18,19]. Information on the COVID-19 vaccination of patients with cancer who are older ≥70 years is mainly based on data extrapolated from recent published studies [15,16,17,18,19]. In a cohort study conducted by Massarweh et al. [15], where the median age of participants was 66 years (range 56–72), 102 patients with solid tumors who were receiving active systemic therapy underwent COVID-19 vaccination. Of these patients, 90% showed an adequate antibody response to the BNT162b2 vaccine. Renor-Riviere [16] reported a retrospective analysis of the safety and efficacy of the BNT162b2 mRNA COVID-19 vaccine in 13 patients with solid tumors; however, the median age of the patients was 17 years (range 16–21). The vaccine was found to be efficacious with a good safety profile. Thakkar et al. [17] reported a high seroconversion rate (94%) in 200 patients with cancer (67% with solid tumors and 33% with hematological malignances) who completed their full vaccination schedule according to the Food and Drug Administration’s guidance. Monin et al. [18] evaluated the safety and immunogenicity of one versus two doses of COVID-19 vaccine BNT162b2 for patients with cancer. This study enrolled 151 patients with cancer: 95 with solid tumors and 56 with hematological malignances. The median age was 73 years (IQR 64.5–79.5). The vaccine was safe and showed adequate immunogenicity. Barriere et al. [19] reported the results of 122 assessable patients with solid tumors undergoing BNT162b2 COVID-19 vaccination; 86% of patients were treated with chemotherapy ± target therapy, and the median age was 69.5 years (range 44–90 years). The immunogenicity after the second dose of the vaccine was adequate and without serious adverse events. Polich et al. [28] evaluated the humoral response in 223 patients with solid tumors after receiving COVID-19 vaccine BNT162b2, where the median age was 67 years (IQR 60–75 years). After the second dose of the vaccine, a high seroconversion rate was observed with good tolerance. Pimpinelli et al. [29] reported the immunogenicity and safety of the BNT162b2 COVID-19 vaccine in patients with hematological cancer. The study included 42 multiple myeloma (MM) patients with a median age of 73 years (range 47–78) and 50 myeloproliferative disease (MPD) patients with a median age of 70 years (range 28–80). The seroprotection rate was 78.6% in MM patients and 88% in MPD patients. Herishanu et al. [30] evaluated the humoral immune response to the BNT162b2 COVID-19 vaccine in 167 patients with chronic lymphocytic leukemia (CLL). The median age was 71 years (range 63.0–76.0). The response rate was highest in patients who obtained clinical remission after treatment, at 79.2%. This vaccination was well tolerated.

Terpos et al. [31] evaluated using neutralizing antibodies (NAbs) against SARS-CoV-2 in 276 patients with plasma cell neoplasms after vaccination with either the BNT162b2 or the AZD1222 vaccine, on days 1 (before the first vaccine shot), 22, and 50. Vaccination with either two doses of the BNT162b2 vaccine or one dose of the AZD1222 vaccine leads to lower production of NAbs in patients with MM compared with controls on days 22 and 50 (*p* < 0.001 for all comparisons). The median age of these patients was 74 years (IQR 62–80), and the vaccination was well tolerated. To our knowledge, only one study described the efficacy and safety of COVID-19 vaccines in older patients with cancer [32]. This study included 36 evaluable patients aged ≥80 years: 10 with hematological malignances and 26 with solid tumors. A total of 86.1% of these patients were on active treatment during vaccination. However, this study has several biases as reported by the authors: the low number of patients who agreed to undergo serological testing after being vaccinated, and the short follow-up about the duration of the serologic response.

More recently, we reported the immunogenicity and safety of BNT162b2 or messenger RNA-1273 vaccines in 257 evaluable patients with solid tumors: the median age was 65 years (range 28–86 years), the seropositivity rate was 75.88%, and no grade 3–4 side effects were reported [33]. Shmueli et al. [34] reported on the efficacy and safety of BNT162b2 vaccination in 129 cancer patients where the median age was 62.4 years (range 32–88), the seropositivity rate was 84.1% after the second dose, and vaccine-related serious adverse events were not observed. Finally, in a recent meta-analysis, we reported that the majority of patients with solid tumors had adequate antibody responses; the patients’ median age ranged from 66 to 82 years. However, in this meta-analysis, there were very few patients older than 70 years [35].

## 5. Conclusions

In our study, 115 cancer patients were older than 70 years; this cohort prospective study showed that 75 (65.22%) cancer patients older than 70 years were seropositive for the SARS-CoV-2 antibody IgG between 15 and 42 days after the second dose of BNT162b2 or mRNA1273 vaccination versus 100% of the control group.

## Figures and Tables

**Table 1:** Patients’ data and the results of anti-COVID-19 vaccination. (BMI: body mass index and IQR: interquartile range).

Variable	Patients *N* = 115 (100%)	Positive Serologic Response *n* = 75 (65.22%)	Negative Serologic Response *n* = 40 (34.78%)	*p*-Value
Median age |IQR| (range) years	73 |72–76| (70–86)	73 (71–76)	74.35 (72–78)	0.52
Sex				
Female *n*(%)	64 (55.65)	46 (71.88)	18 (28.12)	0.09
Male *n*(%)	51 (44.35)	29 (56.86)	22 (43.14)	
BMI (kg/m^2^) median |IQR|	24.08 |21.86–26.70|	23.78 |21.5–27.06|	24.69 |22.68–26.67|	0.29
Stage				
Non-metastatic *n*(%)	40 (34.78)	27 (67.50)	13 (32.50)	0.71
Metastatic *n*(%)	75 (65.22)	48 (64.00)	27 (36.00)	
IgG T1 (AU/mL) median |IQR|	55.5 |4.6–268|	189 |60–280|	3.8 |3.8–5.55|	<0.01
Cancer				
Solid tumors	94 (81.74)	66 (70.21)	28 (29.79)	0.02
Hematological malignances	21 (18.26)	9 (42.86)	12 (57.14)	
Primary solid tumor location				
Gastrointestinal *n*(%)	31 (32.98)	20 (64.52)	11 (35.48)	0.18
Genitourinary *n*(%)	7 (7.45)	4 (57.14)	3 (42.85)	
Breast *n*(%)	19 (20.21)	16 (84.21)	3 (15.79)	
Lung *n*(%)	16 (17.02)	10 (62.50)	6 (37.50)	
Gynecological *n*(%)	8 (8.51)	6 (75.00)	2 (25.00)	
Other *n*(%)	13 (13.83)	10 (76.92)	3 (23.08)	
Active anticancer treatment				
Yes *n*(%)	100 (86.95)	64 (64.00)	36 (36.00)	0.50
No *n*(%)	15 (13.04)	11 (73.33)	4 (26.67)	
Treatment				
Chemotherapy *n*(%)	66 (57.39)	42 (63.64)	24 (36.36)	0.68
Immunotherapy *n*(%)	17 (14.78)	9 (52.94)	8 (47.06)	
Hormone therapy *n*(%)	4 (3.48)	3 (75.00)	1 (25.00)	
Biological therapy *n*(%)	13 (11.30)	10 (76.92)	3 (23.08)	
No treatment *n*(%)	15 (13.04)	11 (73.33)	4 (26.67)	
Anti CD-20	7(6.09)	1 (14.29)	6 (85.71)	0.01
Line *n*(%)				
Neoadiuvant/adiuvant	13 (13.54)	8 (61.54)	5 (38.46)	0.46
I line	51 (53.13)	30 (58.82)	21 (41.18)	
>I line	32 (33.33)	23 (71.88)	9 (28.13)	
Comorbidities				
No *n*(%)	41 (35.65)	25 (60.98)	16 (39.02)	0.48
Yes *n*(%)	74 (64.35)	50 (67.57)	24 (32.43)	
Hypertension *n*(%)	45 (39.13)	28 (62.22)	17 (37.78)	0.69
Diabetes *n*(%)	14 (12.17)	6 (42.86)	8 (57.14)	0.08
Cardiovascular *n*(%)	28 (24.35)	20 (71.43)	8 (28.57)	0.43
Endocrine *n*(%)	12 (10.43)	5 (41.67)	7 (58.33)	0.11
Other *n*(%)	38 (33.04)	21 (55.26)	17 (44.74)	0.12
Laboratory				
Neutrophil count (×10^3^/μL) median|IQR|	3.15 |2.4–4.65|	3.15 |2.36–4.65|	3.23 |2.49–4.49|	0.98
Lymphocyte count (×10^3^/μL) median|IQR|	1.45 |0.9–1.89|	1.5 |0.9–1.97|	1.36 |0.87–1.89|	0.59

## Data Availability

The datasets generated during and/or analyzed during the current study are available from the corresponding author upon reasonable request.
